# The emergence of egocentric biases when comparing others is independent of whether groups or individuals are being compared

**DOI:** 10.1098/rsos.260134

**Published:** 2026-10-07

**Authors:** Marta Rodríguez Aramendía, Raphael Kaplan

**Affiliations:** Department of Basic Psychology, Clinical Psychology and Psychobiology, Universitat Jaume I, Castelló de la Plana, Spain

**Keywords:** social neuroscience, social reference frames, egocentric anchoring-and-adjustment, perspective-taking

## Abstract

Flexible social comparisons require transforming information across reference frames, a process thought to depend on map-like computations in the hippocampal formation. Previous work has shown that egocentric social biases—an implicit reliance on self-knowledge—emerge when relative attributes of others are integrated into absolute social reference frames. However, it remains unclear whether this bias depends on the comparison being made in a specific reference frame (absolute vs relative), or on the type of social agent involved (individuals vs groups). Across two behavioural experiments, we employed a social coordinate transformation task where participants learned a stranger's preferences relative to either an individual or a group (relative frame) and then made inferences within an absolute reference frame defined by the alternative entity type. Across both experiments, egocentric anchoring-and-adjustment was observed during rating, and accuracy correlated with a map-like distance metric in the absolute reference frame. Crucially, self-preferences biased performance across all configurations despite being task-irrelevant. A formal model comparison revealed that the discrepancy between the self and the stranger was the primary predictor of performance, outperforming models based on absolute or relative frame discrepancies alone. These findings indicate that egocentric anchoring is a fundamental feature of flexible social comparisons regardless of social entity.

## 1. Introduction

Social learning is essential for navigating interpersonal interactions and making informed decisions in complex social environments. When forming impressions of others, the meaning of their traits depends on the reference frame used for comparison [1]. For example, someone who ‘enjoys intellectual discussions’ might appear more enthusiastic than you, yet less enthusiastic than a university professor, and still above average relative to the general population. Importantly, these judgements refer to the same underlying preference—what changes is the reference frame against which the individual is evaluated. Because social information is often unpredictable and context-dependent, successful social cognition requires flexibly switching between reference frames.

Self-preferences serve as a reference point when making inferences about others. A growing body of work shows that individuals frequently rely on their own preferences and traits as a starting point when evaluating others, consistent with the anchoring-and-adjustment heuristic [2–4]. Originating in judgement and decision-making research [5], this mechanism describes the tendency to rely on an initial internal estimate (the ‘anchor’) and to adjust only partially from it when integrating new information. In the social domain, this means that inferences about others often begin by projecting one's own preferences, with greater divergence from the self-requiring more cognitive effort [6]. Accordingly, most paradigms studying self–other inferences have focused on dyadic comparisons—directly judging another individual relative to oneself—implicitly treating the self as the primary reference frame for social evaluation.

However, despite substantial work on how people anchor social judgements to the self, very few studies have examined how different social reference frames are integrated. Although the analogy between social and spatial reference frames has long been noted [7,8], most empirical evidence on reference-frame integration comes from spatial cognition. In the spatial domain, egocentric reference frames encode locations relative to the observer, whereas allocentric frames represent spatial relations relative to external landmarks [9]. Successful navigation requires flexibly combining these frames, a process supported by path integration mechanisms that estimate one's position by accumulating distance and direction from a known starting point [10]. Extensive research has shown that specific subregions of the hippocampal formation integrate egocentric and allocentric information during such computations [11,12]. By contrast, whether and how multiple social reference frames are integrated—beyond simple self–other comparisons—is less explored.

This gap exists partly because the meaning of allocentrism within the social domain remains conceptually debated. In social psychology, allocentrism has been traditionally associated with a self-construal orientation in which individuals prioritize others, social norms or collective goals over personal preferences [13]. An alternative interpretation, aligned with spatial cognition, conceptualizes social allocentrism as an absolute social reference frame that encodes relationships among others independently of the self. In this view, allocentric social representations capture how individuals or groups relate to one another within a broader social structure, rather than how they relate to the observer [14]. This framework may support flexible transformations between self-related and other-related perspectives, allowing social knowledge to be organized in a manner analogous to spatial cognitive maps.

Relating social to spatial allocentrism, increasing evidence suggests that abstract knowledge, including social knowledge, is assimilated via cognitive map-like representations in the hippocampal formation [15–23]. Analogy between spatial and social reference systems and the emerging literature on abstract cognitive maps suggest that social inference may also rely on mechanisms capable of flexibly integrating multiple reference frames. Yet only recently have studies begun to investigate how the brain integrates different social reference frames to flexibly navigate in social context [14,24–26]. Following this idea, recent studies have developed social reference-frame transformation tasks [24,25] in which participants first learn a stranger's preferences relative to a particular agent (relative reference frames) and then must judge the stranger's preference in a world-centred frame (absolute reference frames). Crucially, these transformations concern everyday preferences, such as the likelihood of engaging in specific activities. For example, a stranger's preference for intellectual discussion may be encoded relative to a particular agent (e.g. ‘more than a professor’), but must then be mapped onto a common scale that enables direct comparison across agents (e.g. professors, students, or the general population). Judgements made within this common representational space are what we refer to as social comparisons in an absolute reference frame. A key feature of this framework is that it allows the quantification of absolute distance—analogous to physical distance on a spatial map—defined as the numerical separation between agents’ preferences on a common scale and calculable directly from the preference ratings that participants provide for themselves and for other social agents during the task. These tasks require transforming information from a self- or other coordinate system into a more global and inclusive one, providing a powerful approach to investigate how the brain supports reference-frame transformations in the social domain.

Initial evidence for social coordinate transformation comes from Kaplan & Friston [24], who showed that participants integrated information about others into an absolute reference frame and identified hippocampal formation involvement in encoding the absolute distance between social entities. However, because self-knowledge was always explicitly required in their paradigm, they could not determine whether these representations were distorted by egocentric anchoring. More recently, Rodríguez Aramendía *et al.* [25] adapted this task and specifically investigated how the participant self-preferences could be distorting the social reference frame transformation process and where this process is supported in the brain. They demonstrated that map-like representations of abstract social information are susceptible to egocentric anchoring-and-adjustment biases in both the hippocampus (HC) and dorsomedial prefrontal cortex (dmPFC) when making judgements in an absolute reference frame defined by social groups (e.g. big-city dwellers versus people living in rural areas). Critically, however, their design always assigned groups to the absolute reference frame and individuals to the relative frame—leaving open the question of whether the observed bias was driven by the computational demands of the absolute reference frame itself, or by the specific use of groups as reference entities.

To directly test whether egocentric anchoring is tied to the type of social agent or is instead a general property of absolute reference-frame computations, we adapted the design of Rodríguez Aramendía *et al.* [25] across two behavioural experiments. Participants gave likelihood ratings on everyday activities (e.g. eat spicy food, read a book, cycle to work) for themselves, two fictitious individuals and two societal groups (people from urban and rural areas). Next, during the transformation phase, participants inferred a stranger's preference relative to an initial social entity (relative reference frame) and then positioned that stranger within a broader, absolute social structure (absolute reference frame). By systematically swapping the roles of individuals and groups across experiments 1 and 2, we were able to (i) replicate anchoring-and-adjustment effects; (ii) reproduce the relationship between accuracy and metric absolute distance between preferences on the absolute reference frame, independently of whether the common scale was defined by individuals or group; and (iii) identify which reference frames are most sensitive to self-preferences.

In line with our preregistration and prior work, we formulated a primary confirmatory hypothesis regarding the relationship between absolute distance and performance. Specifically, we predicted that the greater distance between the inferred stranger's preference and the available choice options within the absolute reference frame would be associated with higher accuracy. In addition, we conducted exploratory analyses to investigate the influence of the self on social reference-frame transformations. Based on prior work, we expected self-preferences to bias judgements particularly within the absolute reference frame, independently of the type of social entity (individuals or groups). These exploratory analyses aimed to identify which reference frames were most susceptible to egocentric biases, and how this bias was reflected in both reaction times and accuracy during the transformation phase.

## 2. Methods

### 2.1. Participants

Two experiments were conducted at Universitat Jaume I. The hypotheses and design of experiment 1 were documented in a protocol written *a priori* (https://osf.io/gbpd6/), and experiment 2 was preregistered on AsPredicted (no. 190261). In both experiments, participants who did not complete the task or whose performance fell below 60% accuracy were excluded from the analyses. A power analysis indicated that 52 participants were required to detect our hypothesized effect size in experiment 1; 98 participants were recruited and 25 were excluded (25.5%), leaving a final sample of 73 participants (50 female; mean age = 20.94, s.d. = 3.59). For experiment 2, the preregistered target was 51 participants; 56 were recruited and 11 were excluded (19.6%), leaving a final sample of 45 (29 female; mean age = 23.04, s.d. = 4.06). Experiment 2 fell slightly short of its preregistered target owing to exclusions; we note that both targets were derived from power analyses for different statistical tests than those reported here. All analyses reported in this manuscript were conducted on the combined sample of both experiments with Experiment included as a predictor (*n* = 118; see §3).

### 2.2. Task

Stimuli were presented using PsychoPy toolbox (v. 2020.1.1) [27] running in Python 3.9. The task consisted of three distinct phases: anchor phase, transformation phase and rating consistency phase (see figure 1A). After the task, participants were asked to indicate how similar they felt to the two individuals and the two groups, on a Likert scale from 1 to 7 (1 being not similar at all and 7 being very similar). The two experiments were identical in every procedural detail except for the social reference frames used in the transformation phase (see §2.4). In experiment 1, the relative reference frame was an individual and the absolute reference frame was a group (see figure 1C). The rest of the task structure was identical across experiments.

**Figure 1.**
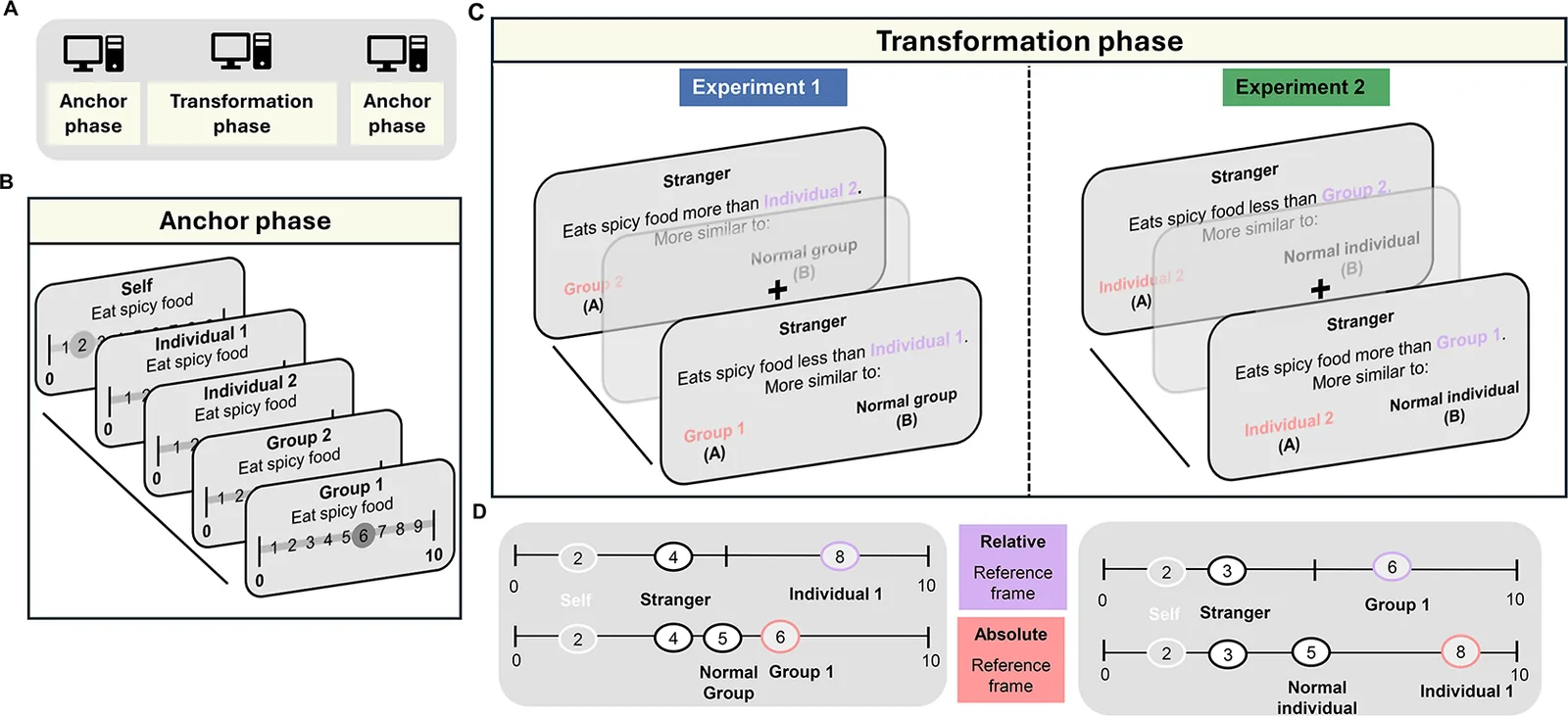
Experimental paradigm. (A) Task overview. The experiment consisted of three sequential phases: an anchor phase, a transformation phase and a rating-consistency (second anchor) phase. The overall structure was identical across both experiments. (B) Anchor phase. Participants first provided likelihood ratings for themselves across everyday scenarios (0–10 scale), followed by confidence ratings (1–5 scale). After reading descriptions of two fictitious individuals and two social groups (city vs village dwellers), participants rated each entity on the same scenarios. The order of entity ratings was counterbalanced across participants. (C) Transformation phase. Participants inferred a stranger's preference relative to a familiar social entity (relative reference frame) and then judged whether this inferred preference was closer to a neutral entity (always fixed at 5) or to one of the two familiar entities defining the absolute reference frame. Left: experiment 1. Participants learned the stranger's preference relative to an individual (relative reference frame) and judged similarity to group preferences (absolute reference frame). Right: experiment 2. The mapping was reversed: participants learned the stranger's preference relative to a group and judged similarity to individual preferences. (D) Reference-frame manipulation. Example ratings for trials for both experiments (experiment 1 on the left and experiment 2 on the right).

### 2.3. Anchor phase

Participants first provided likelihood ratings for 105 everyday activities (adapted from [25]). These ratings were made on a 9-point scale (1–9) embedded within a broader 0–10 range (where 0 indicates ‘impossible’ and 10 indicates ‘extreme likelihood’). This design was specifically chosen to allow for the possibility of a ‘stranger’ having more extreme preferences than the social entities. After each rating, they indicated how confident they were in their response (1–5 scale). To achieve a more varied distribution of self-ratings, we excluded the 30 items closest to each participant's mean, leaving 75 items for subsequent phases.

Then, participants were introduced to two fictitious individuals through brief descriptions, along with the names of two stereotypical groups (‘city dwellers’ and ‘village dwellers’). The individuals were constructed to align more closely with urban or rural lifestyles, and their gender matched that of the participant. Participants could read these descriptions at their own pace. Next, they indicated how likely they thought each social entity would be to engage in all 75 scenarios on the same 0–10 scale, responding within a 10-second time limit (see figure 1B). Preference ratings for each scenario were collected twice: once before and once after the transformation task. In both experiments, the order of ratings during the anchoring phase was counterbalanced such that half of the participants rated individuals before groups, whereas the other half rated groups before individuals.

### 2.4. Transformation phase

The transformation phase required participants to integrate previously learned social information into a new reference-frame structure. Although the trial structure was identical across experiments, the assignment of social entities to relative and absolute reference frames differed between experiments. In experiment 1 (see figure 1C, left), participants inferred a stranger's preference relative to one of the familiar individuals using their previous ratings; and subsequently, in a two-alternative forced choice task (2AFC), they determined whether that stranger's preference is more similar to a neutral group (always a 5), or one of the two groups they previously rated. In experiment 2 (see figure 1C, right), the procedure was reversed. Participants inferred a stranger's preference relative to one of the familiar groups using their previous ratings; and subsequently, they determined whether that stranger's preference is more similar to a neutral individual, or one of the two individuals they previously rated. Accuracy was defined objectively: a response was scored as correct when the participant selected the option whose rating was numerically closer to the stranger's inferred preference, and as incorrect otherwise.

This manipulation allows us to test whether egocentric anchoring-and-adjustment and memory influences social inferences, depending on the type of social entity (individual or group) involved in different reference frame during the task; while inferring the stranger's preferences (relative reference frame) or when comparing the stranger's preferences with the two choice options (absolute reference frame) (see figure 1D).

On each trial, participants were shown a scenario and a cue indicating whether the stranger engaged in that activity ‘more’ or ‘less’ than the reference entity (individual in experiment 1 or group in experiment 2). To compute the stranger preference, participants adjusted the reference entity's rating according to the cue. If the cue was ‘more’, they added half of the difference between the reference entity's rating and the upper end of the scale (10). If the cue was ‘less’, they subtracted half of the difference between the reference entity's rating and the lower end of the scale (0). Participants completed a total of 240 transformation trials, divided into two runs of 120 trials each, with trial order fully randomized. Trials were self-paced, although responses had to be made within a maximum of 10 seconds.

All task-relevant information was displayed simultaneously on the screen throughout each trial. The elements that varied across trials were the relative reference frame, the cue linking the reference entity to the stranger's preference and the two choice options (see figure 1C). Finally, participants completed a consistency phase in which they provided repeated ratings of their own preferences and those of the social entities for the same everyday scenarios.

## 3. Analysis

### 3.1. Behavioural variables

We computed several behavioural variables to test our main hypotheses. First, to assess egocentric anchoring-and-adjustment during the anchoring phase, we calculated rating discrepancy (RD) as the absolute difference between self-ratings and the ratings of the individual or group on each anchoring-phase trial. Second, to examine memory stability, we calculated rating consistency (RC) as the absolute difference between pre- and post-transformation ratings for each entity. These variables were labelled relative or absolute depending on the entity's role in the specific experiment (experiment 1: Relative = Individual, Absolute = Group; experiment 2: Relative = Group, Absolute = Individual). Finally, during the transformation phase, we computed absolute distance (AD), defined as the numerical distance between the stranger's inferred rating and the two choice options presented on each transformation trial.

### 3.2. Anchoring-and-adjustment

To examine anchoring-and-adjustment during the anchoring phase, we fit a linear mixed-effects model (equation (3.1)). Our primary objective was to test whether participants showed stronger egocentric anchoring—that is, larger RD on trials where they took longer to infer others’ preferences. Accordingly, we modelled the relationship between reaction time (RT) and RD and tested whether this association differed depending on whether the anchor entity was an individual or a group,

(3.1) $$RD∼RT_{log}*Entity+\left(1|Participant\right).$$

A positive fixed effect of RT would indicate stronger egocentric anchoring-and-adjustment, such that slower inferences are associated with greater self–other discrepancy. The interaction between RT and Entity tested whether this effect varied as a function of the social entity (individual vs group). We included a random intercept for participants to account for inter-individual variability. Trials without responses in the self-rating or anchoring phase were excluded because RD could not be computed. RTs were log-transformed to correct for right skew, and trials with log-RT values greater than or equal to 2.5 s.d. from the grand mean were removed [28]. This analysis was registered in the OSF project, but we changed the dependent variable and adopted a mixed-modelling approach; we therefore report it as exploratory rather than as a strictly confirmatory test.

### 3.3. Transformation phase and comparison across experiments

We examined how behavioural variables predicted accuracy during transformation trials using a logistic mixed-effects model (equation (3.2)) including RD, RC and AD (see §3.1 for details on the calculation) as predictors for both the relative and absolute reference frames. In experiment 1, individuals served as the relative reference frame and groups as the absolute frame, whereas this mapping was reversed in experiment 2. To test whether the influence of these variables differed between experiments, we included interaction terms between each predictor and experiment. This combined analysis was not preregistered and is reported as exploratory.

(3.2) $$Acc∼\left(AD+RD_{absolute}+RD_{relative}+RC_{absolute}+RC_{relative}\right)\times \;Experiment+\left(1|Participant\right).$$

As preregistered, we additionally tested for a quadratic relationship between absolute rating discrepancy and accuracy by fitting a dedicated model that included linear and quadratic terms for rating discrepancy in the absolute reference frame. We report this dedicated model as the preregistered test of the predicted U-shaped effect. To test whether this effect was robust to the influence of the remaining predictors, we then added the quadratic term to the full model described in equation (3.2).

We note one deviation from the preregistration, the AsPredicted power analysis for experiment 2 was framed around a Spearman rank correlation between absolute distance and accuracy. In the present study, we instead model accuracy with a logistic mixed-effects model.

### 3.4. Effect of anchoring-adjustment bias during transformation trials

During each transformation trial, participants had to keep track of three distinct social entities: the preferences associated with the relative reference frame, the inferred preferences of the stranger and the preferences defining the absolute reference frame. Notably, self-ratings were not required to perform the task correctly.

To determine where self-preferences influenced behaviour during the transformation phase, we fit a set of logistic regression models predicting trial-by-trial accuracy based on different measures of rating discrepancy. We specifically contrasted three competing hypotheses regarding the source of egocentric interference:

— **Relative model**: bias originates from the entity defining the initial relative frame (RD_relative_).
— **Absolute model**: bias originates from the entity defining the target absolute frame (RD_absolute_).
— **Stranger model**: bias originates from the discrepancy between the self and the stranger (RD_self − stranger_).

Model comparison was carried out using the Akaike information criterion (AIC) to formally evaluate relative model fit [29]. All models include absolute distance as a fixed effect to control for the baseline metric distance effect. The models were specified as follows:

(3.3)Relativemodel:Acc∼AD+RDrelative+(1∣Participant),(3.4)Absolutemodel:Acc∼AD+RDabsolute+(1∣Participant),(3.5)Strangermodel:Acc∼AD+RDself−stranger+(1∣Participant),(3.6)Fullmodel:Acc∼AD+RDself−stranger+RDabsolute+RDrelative+(1|Participant).

Generalized linear mixed-effects models were fit using the glmer() function from the lme4 package [30]. All estimated coefficients are reported on the log-odds scale. Continuous predictors were standardized prior to model fitting. Trials in which participants did not respond within the 10-second window during the transformation phase were excluded from analysis. As in previous work [24,25], transformation trials in which both choice options were correct were also omitted. This analysis was not preregistered and is reported as exploratory.

### 3.5. Random effects structure

All models included random intercepts for participants. Although random slopes for within-subject predictors would be the most conservative specification [31], random intercepts only were retained, given the complexity of the design and the number of predictors included. All models were refit with the maximal random effects structure; when convergence failed, random slopes were removed iteratively starting with the lowest-variance predictor. Results were substantively identical and are reported in tables S1–S3 in the supplementary material.

## 4. Results

### 4.1. Anchoring-and-adjustment

To first establish the presence of egocentric anchoring-and-adjustment effects in our task, we analysed behaviour during the anchor phase. During the anchor phase, the average time that participants took to provide ratings of others was 3.06 seconds (s.d. = 1.25 seconds) in experiment 1, and 2.88 seconds (s.d. = 1.15 seconds) in experiment 2. An independent sample *t*-test comparing reaction times across experiments revealed no significant difference, *t*(116) = 1.84, *p* = 0.07. Given that the anchor phase task was identical in both experiments and there were no significant differences in RTs, we combined the data across experiments to increase the statistical power. To study the egocentric anchoring-and-adjustment effect during the anchor phase, how self-rating influences inferences about others, we applied a linear mixed-effects model (equation (3.1)). We observed that the anchor entity, individuals and groups, influenced how participants judged the target. A significant effect of entity was observed (*b* = −0.09, *t*(117) = −6.5, *p* < 0.001), indicating that participants rated the preferences of individual entities closer to their own preferences than those of group targets. Examining the hypothesized positive linear relationship between RT and self versus other discrepancy, we found a significant effect of RT on self–other discrepancy scores (*b* = 0.31, *t*(117) = 6.76, *p* < 0.001). This result suggests that increases in RT correspond to increases in the magnitude of correction away from a self-anchor—evidence of serial adjustment present in anchoring-and-adjustment (figure 2A). Finally, we did not find a significant interaction between RT and entity (*b* = 0.06, *t*(117) = 1.63, *p* = 0.10), suggesting that we did not find evidence for a difference between individuals and groups. Together, these results confirm the presence of anchoring-and-adjustment processes in both experiments during the rating phase.

**Figure 2.**
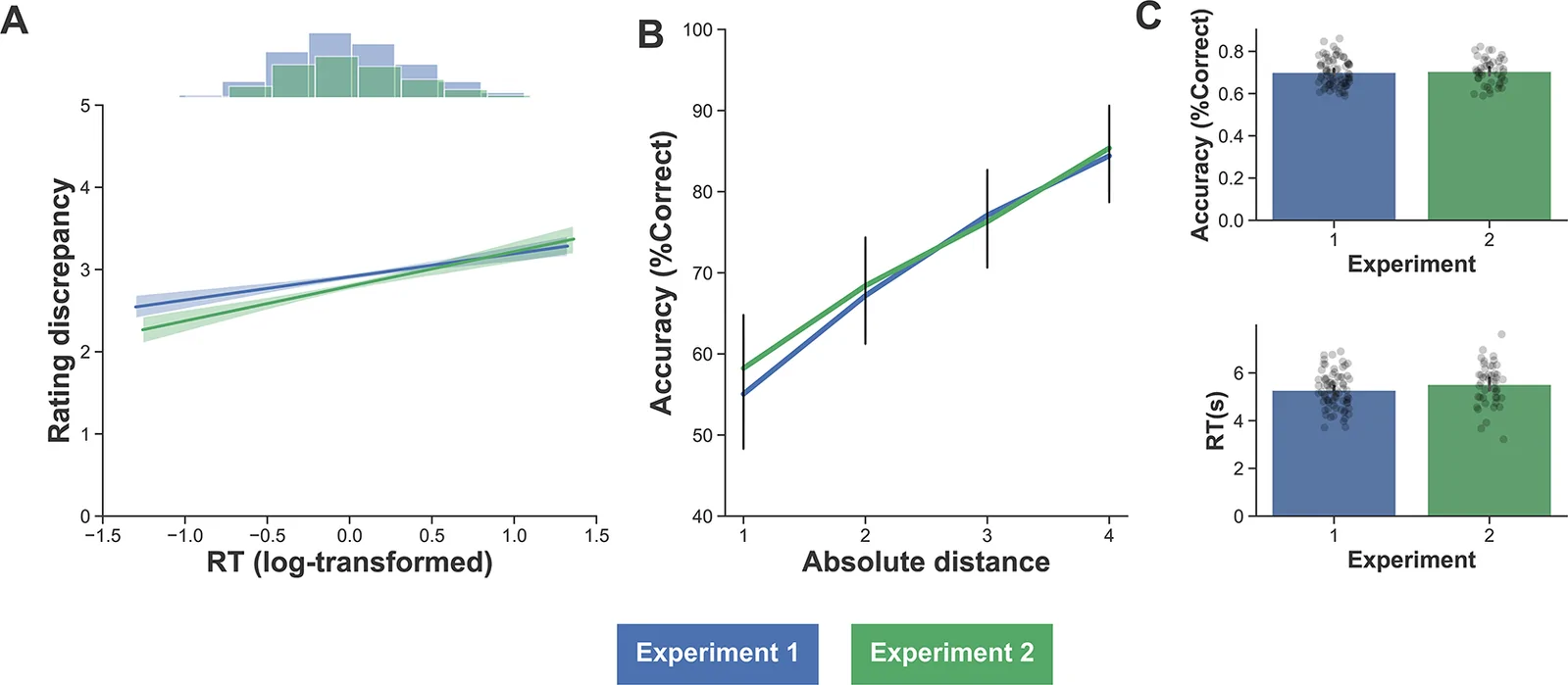
Replication analyses. (A) Anchoring-and-adjustment effect. Relationship between reaction time (RT) and rating discrepancy. Each line represents one experiment. In both experiments, longer RTs were associated with larger self–other discrepancies. The *x*-axis shows RTs centred within participants. (B) Accuracy as a function of absolute distance (AD). Higher AD between the stranger's inferred preference and the two choice options increased the probability of a correct response in both experiments. (C) Comparison of overall task performance across experiments. No significant differences were found in accuracy (top; *t*(116) =   − 0.39, *p*  =  0.7) or reaction times (*t*(116)  =   − 1.60, *p*  =  0.112).

### 4.2. Memory and rating discrepancy

To characterize how memory precision and self–other discrepancy vary across social targets, and to assess whether these patterns differ between experiments, we analysed RC and RD (see §3.1 for calculation). A mixed ANOVA revealed no significant interaction between experiment and social entity on consistency (F_2,232_ = 0.09, *p* > 0.05), indicating that the pattern was comparable across experiments. However, we observed a robust main effect of social entity (F_2,232_ = 34.75, *p* < 0.001, partial eta squared $\eta_{p}^{2}=\;0.23\varepsilon )$. Post hoc comparisons showed that participants were most consistent when rating their own preferences, less consistent when rating group preferences and least consistent when rating individual preferences (figure 3A). This ordering was highly consistent across both experiments, with a clear advantage for groups over individuals.

**Figure 3.**
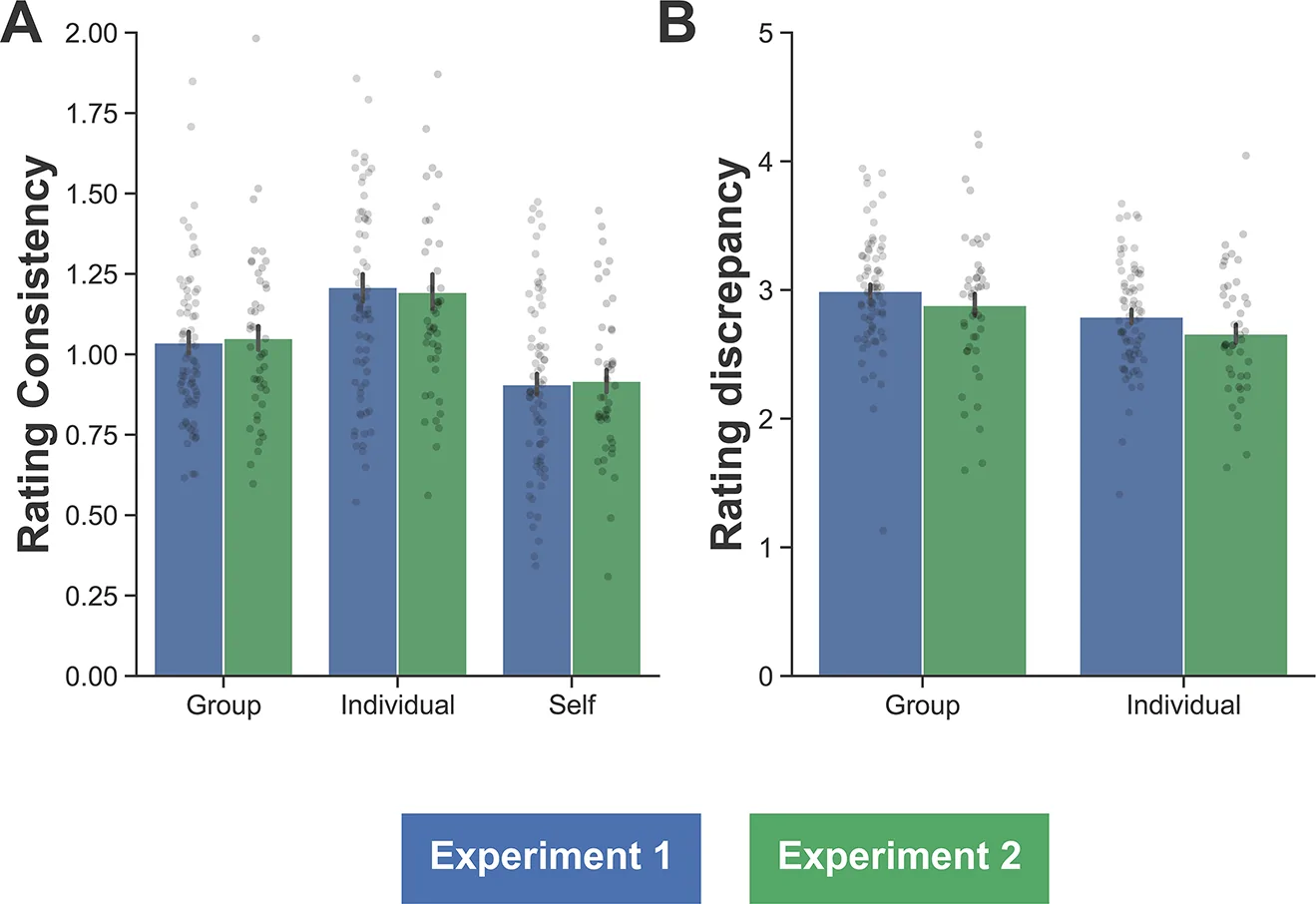
Memory and rating discrepancy across social entities. (A) Rating consistency (RC). There was no interaction between experiment and social entity (F_2,232_  =  0.09, *p* > 0.05), but a robust main effect of social entity (F_2,232_  =  34.75, *p* < 0.001, $\eta_{p}^{2}=\;0.23\varepsilon$). Participants showed highest consistency for self-ratings, followed by group ratings and lowest consistency for individual ratings. (B) Rating discrepancy (RD). No interaction between experiment and social entity was observed (F_1,116_  =  0.13, *p* > 0.05), but a strong main effect of social entity (F_1,116_  =  52.43, *p* < 0.001, $\eta_{p}^{2}=\;0.31\varepsilon$). Group preferences were perceived as more dissimilar from the self than individual preferences. Each dot represents one participant.

Next, we studied how closely participants felt their own preferences matched those of the social target, rating discrepancy for each reference frame (figure 3B). A mixed ANOVA revealed no significant interaction between experiment and social entity on discrepancy (F_1,116_ = 0.13, *p* > 0.05), indicating that the pattern was comparable across experiments. However, we observed a robust main effect of social entity (F_1,116_ = 52.43, *p* < 0.001, $\eta_{p}^{2}=\;0.31\varepsilon$) where groups preferences were consistently rated as more dissimilar to the participants’ own preferences than the individual's preferences (*t*(117) = 7.26, *p*  <  0.001; figure 3A).

Together, these results indicate that both RC and RD vary systematically across social targets, but do so consistently across experiments. This provided supporting evidence to include these comparable variables in subsequent analyses.

### 4.3. Transformation phase and comparison across experiments

To test our primary preregistered hypothesis regarding the effect of absolute distance on performance, we first examined the relationship between accuracy and absolute distance. Consistent with previous work, we observed a positive relationship between accuracy and the absolute distance between the inferred stranger's rating and the two choice options in both experiments (figure 2B).

Before modelling trial-level effects, we assessed whether overall performance was comparable across experiments. Independent samples *t*-tests revealed no significant difference in accuracy between experiments 1 and 2, *t*(116) = −0.39, *p* = 0.700, nor in reaction times, *t*(116) = −1.60, *p* = 0.112 (see figure 2C).

We then fit a logistic mixed-effects model to test if the effect of absolute distance held when accounting for other variables, while also exploring if there were other additional influences on performance. We also test whether these effects differed across experiments. The model included absolute distance, rating discrepancy and rating consistency as predictors in both the relative and absolute reference frames, with Experiment included to capture overall differences between experimental conditions and to test if the effects of the behavioural variables were modulated by the type of social entity (individual vs group). Unlike the preregistered test of absolute distance, this full model was not preregistered and is reported as exploratory.

The results of the logistic mixed-effects model are presented in table 1. Significant main effects were observed for most predictors, except for RC_relative_ and Experiment. In accordance with our primary preregistered hypothesis, we observed a robust absolute distance effect: the relationship between AD and accuracy was significant and positive (estimate = 0.55, s.e. = 0.045, *z* = 12.30, *p*  < 0.001), indicating that larger distances between the choice options and the stranger's preference increased the probability of a correct response (figure 2B). Both RD_absolute_ and RD_relative_ also showed significant positive effects (RD_absolute_: estimate = 0.100, s.e. = 0.047, *z* = 2.10, *p* = 0.035; RD_relative_: estimate = 0.157, s.e. = 0.046, *z* = 3.40, *p* = 0.001), such that greater discrepancies between self and others’ preferences—whether for groups or individuals—were associated with higher accuracy. By contrast, RC_absolute_ showed a significant negative effect (estimate = −0.348, s.e. = 0.042, *z* = −8.31, *p*  < 0.001), suggesting that poorer memory for ratings in the absolute reference frame was associated with lower performance. No significant effect was found for RC_relative_ (estimate = 0.022, s.e. = 0.044, *z* = 0.52, *p* = 0.604). Finally, the Experiment predictor did not reveal any significant differences (estimate = 0.014, s.e. = 0.064, *z* = 0.21, *p* = 0.83) in overall performance between experiments (see figure 2C).

**Table 1.** Fixed effects from the logistic mixed-effects model comparing accuracy across experiments. Main effects were significant for most predictors except RC_relative_ and experiment. The interaction RD_relative_ and experiment was significant, suggesting that relative reference frame discrepancy affects performance differently between experiments. AD = absolute distance, RD = rating discrepancy, RC = rating consistency. Asterisks showing significant differences: * = *p* < 0.05, ** = *p* < 0.01, *** = *p* < 0.001.

fixed effects	estimate	s.e.	*z* value	*p* value
(intercept)	0.932	0.094	9.90	< 0.001***
AD	0.556	0.045	12.30	< 0.001***
RD_absolute_	0.100	0.047	2.10	0.035*
RD_relative_	0.157	0.046	3.40	0.001***
RC_absolute_	−0.348	0.042	−8.31	< 0.001**
RC_relative_	0.022	0.044	0.52	0.604
experiment	0.014	0.064	0.21	0.830
AD: experiment	−0.051	0.031	−1.66	0.096
RD_absolute_: experiment	−0.041	0.032	−1.27	0.204
RD_relative_: experiment	−0.087	0.031	−2.79	0.005**
RC_absolute_: experiment	0.008	0.028	0.29	0.773
RC_relative_: experiment	−0.017	0.029	−0.58	0.566

The interaction between our behavioural predictors and experiment allow us to see if the effect of these predictors were modulated by the type of social entity (individual/group). We did not observe significant interactions for AD (estimate = −0.051, s.e. = 0.031, *z* = −1.66, *p* = 0.096), RD_absolute_ (estimate = −0.041, s.e. = 0.032, *z* = −1.27, *p* = 0.204), RC_absolute_ (estimate = 0.008, s.e. = 0.028, *z* = 0.29, *p* = 0.773) and RC_relative_ (estimate = −0.017, s.e. = 0.029, *z* = −0.58, *p* = 0.566). However, we observed a significant interaction between RD_relative_ and experiment (estimate = −0.087, s.e. =  0.031, *z*  = −2.79, *p* = 0.005). This indicates that the positive effect of relative discrepancy on accuracy was weaker in experiment 2 than in experiment 1. While higher RD_relative_ values predicted greater accuracy in both experiments, the strength of this relationship decreased when the relative reference frame corresponded to the group rather than the individual.

Although this cross-experiment comparison was not preregistered, the results indicate that the effect of absolute distance on performance is consistent across experiments, supporting the idea that this computation does not depend on the type of social target defining the reference frame.

As preregistered, we tested for a quadratic effect of absolute rating discrepancy on accuracy. In a dedicated model containing only linear and quadratic terms for absolute rating discrepancy, the quadratic term was significant and positive (*b* = 0.46, *z* = 8.82, *p* < 0.001), consistent with the predicted U-shape. However, this effect was confounded with absolute distance: once absolute distance and the remaining predictors were included, the quadratic term dropped to near zero and was no longer significant (*b* = 0.004, *z* = 0.07, *p* = 0.95). We therefore retain the linear specification in our primary model and interpret the preregistered quadratic effect as lacking robustness to control for absolute distance.

### 4.4. Self-rating during transformation trials

Finally, to determine in which reference frame self-related information influences performance during social transformations, and to test our preregistered hypothesis that self-preferences would influence performance even when task-irrelevant, we conducted a formal model comparison. We fit several logistic models and selected the model that best explained the data, using AIC. All models included absolute distance between the two choice options, together with a self–other discrepancy defined either in the absolute reference frame (absolute model), the relative reference frame (relative model) or the self–stranger discrepancy (stranger model). A full model including all three discrepancy measures was also tested. Formal equation provided in §3.4.

Model comparison indicated that the stranger model provided the best fit to the data, as shown in table 2, outperforming all alternative specifications. This suggests that participants’ performance during the transformation phase was most strongly driven by the discrepancy between their own ratings and those of the stranger, rather than by discrepancies based on absolute or relative reference frames alone.

**Table 2.** Model comparison results for transformation trials using AIC and Bayesian information criterion (BIC). Lower values indicate better model fit. The stranger model outperformed all alternatives.

model	AIC	BIC
relative model	30 189.71	30 222.38
absolute model	30 195.40	30 228.07
**stranger model**	**30 180.43**	**30 213.11**
full model	30 183.51	30 232.52

To examine the direction of the effects, we analysed the estimates from the stranger model. This analysis revealed a robust positive effect of absolute distance on accuracy (estimate = 0.514, s.e. = 0.015, *p* < 0.001), such that larger distances between the stranger's inferred rating and the two choice options increased the likelihood of a correct response. We also observed a significant positive effect of RD_self-stranger_ (estimate = 0.056, s.e. = 0.017, *p* = 0.001), indicating that greater differences between participants’ own ratings and those of the stranger were associated with higher accuracy (see table 3).

**Table 3.** Fixed effects from the logistic mixed-effects stranger model. Stranger model included absolute distance (AD) and self–stranger rating discrepancy (RD_self-stranger_) as predictors of accuracy during transformation trials. Both predictors showed significant positive effects, indicating that larger distances between choice options and greater self–stranger discrepancy increased the probability of a correct response. Coefficients are reported on the log-odds scale. Asterisks showing significant differences: * = p < 0.05, ** = p < 0.01, *** = p < 0.001.

fixed effects	estimate	s.e.	*z* value	*p* value
intercept	0.932	0.030	30.61	< 0.001***
AD	0.514	0.015	34.88	< 0.001***
RD_self-stranger_	0.056	0.017	3.23	0.001**

To assess whether this effect was also reflected in RTs, we fit the same model using RTs as the dependent variable. No significant effect of RD_self-stranger_ on RTs was found (estimate = −0.002, s.e. = 0.003, *p* = 0.52). However, absolute distance showed a significant effect on RTs (estimate = −0.01, s.e. = 0.002, *p* < 0.001), with larger distances associated with faster responses.

Together, these results indicate that egocentric anchoring selectively influences the inferred stranger, rather than the reference frames themselves. We note, however, that this analysis was exploratory and not preregistered.

## 5. Discussion

In this study, we asked whether egocentric anchoring-and-adjustment biases during social preference transformation tasks are influenced by the positions that different social agents (individuals vs groups) occupy within relative and absolute reference frames. As a first step, we examined the presence of anchoring bias and replicated previous work showing that participants reliably exhibit anchoring-and-adjustment when inferring others’ preferences [4,25,32]: longer response times were associated with greater adjustments away from self-anchors (figure 2A). We also found a positive relationship between the absolute distance between the two choice options and the stranger, independent of whether the comparison involved individuals or groups within the absolute reference frame (figure 2B). Additionally, the distance between participants’ own ratings and the stranger's rating influenced performance, even though self-preferences were not required to perform the task (table 3). We cover the implications of these results and how they build on previous literature.

A key question was whether egocentric anchoring would distort reference frame transformations differently depending on whether the reference entities were individuals or groups. We found a remarkably consistent pattern: self-related information influenced the transformation process in both experiments and across both reference frames. This indicates that egocentric bias is not tied to the type of social agent, but reflects a broader tendency to rely on self-preferences even when they are not required for computations. This finding aligns with established theories of anchoring-and-adjustment, which posit that the self frequently serves as the primary reference point during social and memory-guided inferences [33,34].

The persistent influence of self-related information aligns with the idea of self–other merge, as described in earlier studies [35,36], where self- and other-related representations overlap to some degree, making it difficult for participants to fully suppress egocentric information during decision-making. An additional mechanism that may contribute to this effect is the self-reference effect [37,38] where self-ratings were remembered with higher fidelity than other ratings, which probably strengthened the impact of self-preference during transformation trials. Supporting this interpretation, recent evidence has implicated the ventromedial prefrontal cortex (vmPFC) in maintaining the stability and consistency of self-related representations: patients with vmPFC damage show reduced consistency in self-related judgements relative to judgements about others, compared with healthy control participants [39]. Notably, we found that group ratings were remembered more accurately than individual ratings, suggesting that groups may serve as more stable reference points in social cognition [40].

Moreover, the direction of our egocentric anchoring effect in which larger discrepancies between self and other ratings were associated with better performance contrasts with findings in emotional prediction tasks. In emotional prediction tasks, egocentric projection—the tendency to use one's own emotional state as a proxy for others—has been shown to improve predictive accuracy [41]. Conversely, reliance on self-related information in our paradigm appears to be maladaptive when it increases confusion between self and others [42], where it is helpful only when self and social agents preferences are clearly distinct.

A robust influence of absolute distance also emerged during the social transformation task. This supports the idea that participants relied on distance metric information to guide social inferences, consistent with hippocampal distance-coding during similar tasks [24]. More broadly, this behavioural pattern aligns with growing evidence that social and spatial cognition rely on shared computational mechanisms, in which social inferences are supported by cognitive-map-like representations in hippocampal–entorhinal circuits [18,43,44]. Consistent with this spatial view, recent work shows that mentalizing spans multiple distinct dimensions—mental states, personality and intelligence—pointing to a geometry-like structure in how we represent other minds [45], further supporting the idea that social and spatial representations may share a common geometric organization.

Our behavioural findings also align closely with the neural mechanisms reported by Rodríguez Aramendía *et al.* [25]. In that study, both the dmPFC and the hippocampal–entorhinal system represented egocentric anchoring biases during social inference, although their design examined this effect only when groups occupied the absolute reference frame. The present results extend these findings by using a larger sample and two independent experiments, demonstrating that the anchoring bias effect is behaviourally robust across different social agents (individuals vs groups) positioned in either relative or absolute reference frames. Although the current study does not include neuroimaging data, the behavioural patterns we observe strongly suggest that similar neural mechanisms would operate when individuals, rather than groups, define the absolute reference frame. This interpretation is consistent with the proposal that the hippocampal formation integrates multiple social reference frames [14,24,25] and performs transformations analogous to those observed in spatial cognition [11].

The task required participants to maintain three different social representations in mind (individual, groups and stranger). Importantly, although this introduces a working memory and computational demand, this requirement was constant across trials, making it unlikely to explain the selective influence of self-ratings observed in our results. Across our analyses, we examined which of these social agents was most influenced by self-rating. The results showed that self-ratings had the strongest influence on the stranger's preferences, rather than on the social entity defining the absolute or relative reference frames. One possible interpretation of this pattern is that anchoring serves as an uncertainty-reduction mechanism [34]: when inferring the preferences of an unfamiliar individual, participants may rely more heavily on the information provided by their own preferences. From this perspective, the stranger represents the most uncertain social entity, making it particularly susceptible to egocentric anchoring.

Future research should investigate whether egocentric anchoring represents a domain-general computational bias that transfers between social and spatial cognition. While previous studies have explored the connection between social and spatial perspective taking [46], a clear consensus has yet to emerge. Our findings provide a robust behavioural foundation to test whether egocentrism acts as a stable latent trait; specifically, whether individuals who exhibit heightened egocentric interference during social coordinate transformations also demonstrate parallel distortions in spatial navigation tasks. Computational modelling could disambiguate the effect of self during reference frames transformation and provide insights about how the self distorts social and spatial representations and whether this distortion is similar between both domains. In this context, it is worth noting that while our framework draws primarily on analogies from spatial cognition, the concept of the altercentric reference frame—encoding information relative to another specific person rather than the self—has a long tradition in social cognition research [34,47]. Unifying these two traditions, by integrating egocentric, allocentric and altercentric frames within a common computational framework, represents an important and exciting direction for future research.

The focus of this article was to demonstrate how the self affects reference frame transformation of social agent's preferences into an absolute reference frame, thereby bridging social and spatial domains. Still, we acknowledge that several important social factors were not addressed in the present study. These include the degree of self-similarity [4,34] with social agents, the sense of belonging of participants to the social groups and the role of self-malleability [42] in shaping how we represent and remember others and ourselves. Future work could investigate whether the representation of the social entities changes dynamically across the transformation phase and how self-ratings may influence these representational shifts.

Together, these findings demonstrate that egocentric anchoring acts as a robust feature of social preference transformation, operating across different social agents and reference frames; independently of whether reference entities were individuals or groups. Our findings indicate that self-related information exerts a pervasive influence even when it is not required for task performance, suggesting that the self distorts social transformations. More specifically, this convergence suggests that social and spatial transformations may share nonlinear self-centred transformation mechanisms [26], similar to those observed in spatial navigation, where representations are distorted as a function of distance and orientation relative to the self.

## Supplementary Material



## Data Availability

Data and relevant code for this research work are stored in GitHub https://github.com/MartaRodr/EgocentricBias_TransformingReferenceFrame and have been archived within the Zenodo repository: https://doi.org/10.5281/zenodo.22112886. The repository contains pre-processed behavioural data and all scripts used for statistical analyses and figure generation [48]. Supplementary material is available online [49].
