# Supplementary material for: The emergence of egocentric biases when comparing others is independent of whether groups or individuals are being compared
Source: R Soc Open Sci. 2026 Oct 7;13(10):260134. doi: 10.1098/rsos.260134 (PMC13641921; doi:10.1098/rsos.260134)
Supplement: Supplementary file 1 [file rsos.260134.zip › rsos.260134/rsos260134_si_001.docx]

**Supplementary Tables. Robustness of main results to random effects specification.**

Following recommendations for random effects specification (Barr et al., 2013), all models reported in the main text were refit with the maximal random effects structure, including random intercepts and random slopes for all within-subject predictors. When the maximal structure failed to converge, random slopes were removed iteratively, starting with the predictor showing the lowest variance, until convergence was achieved. Estimated coefficients, standard errors, z-values, and p-values are shown. Results are substantively identical across both specifications.

| **Fixed effects** | **Estimate** | **Std. Error** | **z value** | **p value** |
| --- | --- | --- | --- | --- |
| (Intercept) | 0.95 | 0.10 | 9.25 | $<0.001$*** |
| AD | 0.57 | 0.07 | 8.03 | $<0.001$*** |
| ${RD}_{absolute}$ | 0.10 | 0.05 | 2.12 | 0.034* |
| ${RD}_{relative}$ | 0.15 | 0.046 | 3.42 | 0.001*** |
| ${RC}_{absolute}$ | -0.35 | 0.061 | -5.66 | $<0.001$*** |
| ${RC}_{relative}$ | 0.028 | 0.044 | 0.65 | 0.50 |
| Experiment | 0.015 | 0.07 | 0.22 | 0.83 |
| AD: Experiment | -0.045 | 0.05 | -0.93 | 0.35. |
| ${RD}_{absolute}$: Experiment | -0.040 | 0.032 | -1.25 | 0.211 |
| ${RD}_{relative}$: Experiment | -0.087 | 0.031 | -2.77 | 0.005** |
| ${RC}_{absolute}$: Experiment | 0.008 | 0.042 | 0.20 | 0.84 |
| ${RC}_{relative}$: Experiment | -0.022 | 0.03 | -0.76 | 0.44 |

**Supplementary Table S1.** Replication of the main accuracy model (Equation 2) with an expanded random effects structure. Although the maximal random effects structure was attempted, convergence was only achieved with random slopes for AD and RC absolute. Results are consistent with the main text, confirming that the random intercepts-only specification does not affect conclusions. AD = Absolute Distance, RD = Rating Discrepancy, RC = Rating Consistency.

| **Model** | **AIC** | **BIC** |
| --- | --- | --- |
| Relative Model | 30103.85 | 30177.37 |
| Absolute Model | 30108.22 | 30181.73 |
| **Stranger Model** | **30089.92** | **30163.44** |
| Full Model | 30094.11 | 30257.48 |

**Supplementary Table S2.** Model comparison results for transformation trials (Equations 3–6) replicating the analysis reported in the main text with the maximal random effects structure, including random intercepts and random slopes for all within-subject predictors. Lower AIC and BIC values indicate better model fit. The Stranger Model outperformed all alternatives, consistent with the main text findings.

| **Fixed effects** | **Estimate** | **Std. Error** | **z value** | **p value** |
| --- | --- | --- | --- | --- |
| Intercept | 0.932 | 0.030 | 30.66 | $<0.001$*** |
| AD | 0.514 | 0.015 | 34.96 | $<0.001$*** |
| RD_self-stranger_ | 0.056 | 0.017 | 3.25 | 0.001** |

**Supplementary Table S3.** Replication of the Stranger Model (Equation 5) reported in the main text with the maximal random effects structure, including random intercepts and random slopes for all within-subject predictors. Both absolute distance (AD) and self–stranger rating discrepancy (RDself-stranger) remained significant positive predictors of accuracy, consistent with the main text findings. Coefficients are reported on the log-odds scale. AD = Absolute Distance, RD = Rating Discrepancy.

**Description individuals***

[Name] drives quite often but is not very used to everyday traffic. [He/She] knows most of [his/her] neighbours and usually says hello when walking into a public place. [He/She] lives relatively close to nature.

[Name] is used to traffic and often takes public transport to get to work or university. In [his/her] free time, [he/she] likes going to different kinds of restaurants and sometimes orders takeaway.

**All stimuli were presented in Spanish; English translations are provided here for illustration only. Target names were matched to each participant's gender.*
